# Sampling design and estimation procedure for energy audit and carbon footprints for onion crop in India

**DOI:** 10.1038/s41598-025-96054-y

**Published:** 2025-11-10

**Authors:** Kaustav Aditya, Bharti, Pankaj Das, Rahul Banerjee, A. Carolin Rathinakumari, Tauqueer Ahmad, Vinod Kumar Bhargav, G. Senthil Kumaran, S. A. Venu

**Affiliations:** 1 ICAR-Indian Agricultural Statistics Research Institute, New Delhi, India; 2 ICAR-Indian Institute of Horticultural Research, Bengaluru, India; 3 ICAR-Central Institute of Agricultural Engineering, Bhopal, India

**Keywords:** Energy use pattern, Survey weighted estimates, Data envelopment analysis, Renewable energy, Environmental sciences, Mathematics and computing, Statistics, Energy and society, Environmental impact

## Abstract

Agriculture significantly contributes to greenhouse gas emissions, necessitating swift policy action to mitigate its environmental impact, aligning with UN sustainable development Goals (SDGs). Assessing energy usage and targeting energy-intensive operations are key to effective energy management that enables farmers to implement strategies, reducing costs and carbon footprint. Implementing energy audits in agriculture requires a proper sampling methodology to identify energy-intensive operations and explore renewable energy sources. Therefore, the study presents a detailed sampling design and methodology for estimating energy usage in agricultural crops. It outlines sample size determination, allocation across strata, selection and parameter estimation procedures. Non-parametric data envelopment analysis (DEA) identified 7% of farm households as efficiently using energy, with an average technical efficiency of 0.77 for inefficient ones, suggesting a potential 23% resource saving without yield reduction. Additionally, CO_2_ emissions per crop production process highlight the urgency of policy formulation to promote renewable energy sources.

## Introduction

Agricultural system requires energy (direct as well as indirect) as an input at all stages of agricultural production. Direct energy requirements refer to the gross energy of the fuels directly used in the production process. Indirect energy requirements include all remaining processes needed for the production of the input and its use at the farm, including fuel production and transport, raw materials production and transport, energy embedded in equipment and transport of finished products up to the farm. Agriculture globally contributed about 11% of the total global Greenhouse Gas (GHG) emissions^1^. According to the World Health Organization (WHO) Global Strategy on Health, Environment, and Climate Change (2020), environmental quality deteriorated due to the huge emission of CO_2_ and other greenhouse gases. India accounts for around 6.6% of total global CO_2_ emissions in the world, making it the third-largest CO_2_ emitting country after China and the USA (Global Carbon Budget^2^). In India, there is still a wide range of processes that use conventional energy sources as their main source of energy. The IRENA 2013^3^ report also found that at least 700 to more than 800 million people mostly use conventional energy sources as their main source of energy, but the estimates of their use are not accounted for in official statistics. Estimation of direct and indirect energy use is mandatory when elaborating on energy use and energy efficiency in production agriculture, to avoid sub-optimization and incorrect recommendations on technology use in farming operations. In this regard, energy audit in production agriculture is very much important to achieve higher efficiency with minimum energy input. Energy audit in farm operations will provide the benefit of minimum use of energy sources and likelihood of installing energy savings recommendations.

Developing an effective sampling plan for a survey is paramount as it forms the cornerstone for acquiring representative data, which is fundamental for making precise conclusions and facilitating well-informed decisions. A meticulously designed sampling plan ensure that the selected sample accurately mirrors the population under study, thus mitigating biases and strengthening the applicability of findings. A robust sampling plan is indispensable for safeguarding the integrity and validity of survey outcomes, thereby enhancing the reliability and practicality of research findings. Pathak et al. 2014^1^ had also emphasized the development of methodologies/models for quantifying energy use pattern and GHG emissions in agriculture along with exploring mitigation strategies for climate change in India. Estimates of energy inputs consumed for a crop throughout a season can identify operations where energy savings could be realized by changing applied practices to increase the energy ratio. In line with these objectives, the study aims to develop a sampling design and estimation procedures in detail for energy audit survey in agricultural crops in India. A pilot survey was also conducted to generate survey weighted estimates of energy use in onion production in the Karnataka state of India, using proposed sampling methodology. The study also utilized Data Envelopment Analysis (DEA) to identify the most energy-consuming operations and management practices, assess technical and scale efficiencies of the farm households and identify inefficient farm households that were wasting resources. Additionally, the study focused on estimating the amount of CO_2_ emission associated with each stage of onion production in Karnataka, aiming to identify the most energy-consuming practices. The ultimate objective of the study was to propose policy recommendations that encourage the adoption of renewable energy sources and more efficient farming practices to reduce both energy consumption and carbon footprints.

### Review of literature

This section gives an overview of the literature on energy use patterns and Data Envelopment Analysis (DEA), with particular focus on agricultural crop. This review highlights the interest of researchers in increasing efficiency and sustainability of agricultural practices, emphasizing the need of optimizing both energy consumption and resource allocation in onion farming.

Numerous research studies worldwide have examined the energy use efficiency of agricultural crops such as onion^4–6^, vegetables^7–14^, cotton^15^, sunflower^16–18^, legume^19^, cereals^20–22^, fruits such as apricot, pear, cherry, strawberry, grape, peach, nectarine, pomegranate, apple etc.^23–36^. Main indirect energy consumption in onion was in the form of fertilizers^6,37–39^; pumping of irrigation water^6^, human labour energy^55^ etc.

DEA measures the efficiency of a series of Decision-Making Units (DMUs) using linear programming models^49^. Kyrgiakos et al.^40^ investigated the application of DEA models in agriculture, revealing that the majority of the results were derived from traditional DEA models such as the CCR (CRS) and BCC (VRS). Notably, nearly half of the studies examined (46%) utilized both the CRS and VRS models, along with scale efficiency measurements, while 23% applied only the VRS model, and 9% relied solely on the CRS model. While the choice between CRS and VRS models is problem-specific, the VRS assumption is typically preferred in the agricultural sector. This is because increasing inputs does not always lead to a proportional increase in outputs. For example, doubling the amount of fertilizer does not necessarily result in double the production by the end of the cultivation period. CRS scores are mainly used for calculating scale efficiency. Many researchers have conducted studies on Data Envelopment Analysis (DEA) to evaluate the performance of various crops, helping to identify the best-performing farms and agricultural practices^41–45^. By analyzing multiple inputs and outputs, DEA can suggest optimal input combinations to maximize crop yield while minimizing resource overuse.

Although numerous studies have examined energy use efficiency and Data Envelopment Analysis (DEA) in agriculture, fewer studies have focused on developing appropriate sampling designs for data collection and estimation procedures for assessing energy use patterns. A well-structured sampling design is crucial for conducting surveys to ensure that the sample accurately represents the target population. Therefore, this study primarily focused on developing a suitable sampling design and estimation procedure for analyzing energy use patterns. To test the applicability of this design, a survey was conducted on energy use patterns for onion crops in the Karnataka state of India. In addition, DEA was used to identify the most energy-intensive operations, with the goal of replacing these with renewable energy sources

## Materials and methods

### Survey design and sample size

An appropriate sampling strategy is inevitable in order to implement energy audit survey to collect data about energy use in systematic manner. A stratified two-stage sampling design has been proposed to collect data on energy audit surveys in agricultural crops. In proposed sampling design, agro-climatic zones (ACZs) within the state, where the crop of interest holds substantial acreage, were identified as strata. For instance, Karnataka encompasses ten agro-climatic zones, out of which five have significant area under onion cultivation. Therefore, these five agro-climatic zones were considered as strata for conducting an energy audit survey of the onion crop. To select the farm households for the study, a complex stratified two-stage random sampling design was used^46,47^. In this sampling design, the agro-climatic zones were considered as strata, and the villages within each stratum were considered as the first-stage unit (FSU), while the farm households within each village were considered as the second-stage unit (SSU). A sample of 30 villages was allocated to the five strata (ACZs) in proportion to the area of cultivation under the onion crop, based on resource availability and other relevant factors.

$$\text{Number of villages to be selected from }i-\text{th ACZ}= \frac{\text{Area of the onion crop in the } i-\text{th ACZ}}{\text{Area of the onion crop in all selected ACZ}} \times 30$$

**Fig. 1:**
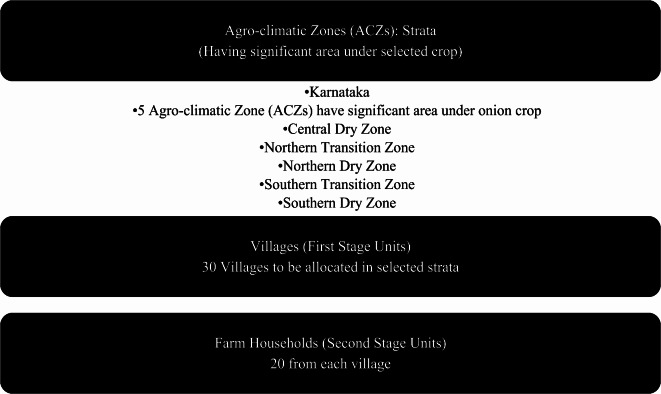
Flowchart of selection of farm households through Stratified Two-stage Sampling.

Further, a frame of the eligible farm households was formulated based on the available land records of the village. From the sampling frame, 20 eligible farms/farm households were selected using equal probability without replacement sampling design. Thus, a total of 600 farm households were selected for energy auditing in the onion crop of the Karnataka state. The sample sizes were determined based on the principle of optimization with respect to the given cost of the survey^46^. A flowchart of proposed sampling design is depicted in Fig. 1.

### Data collection

The data were collected using three questionnaires designed at the institute. Questionnaire-I include the data on (i) basic information about the selected village, and (ii) farm household-wise information. Questionnaire-II involved the re-tabulation of data from Questionnaire-I, specifically the list of operational holdings in the selected village. Lastly, Questionnaire-III was designed for the detailed operational holding survey.

### Estimation procedure as per proposed sampling design and data analysis

Estimates of energy consumption for various farm operations based on collected field data, were calculated as:

The estimates of average value of the variables in the selected ACZ are as:

$${\widehat{\overline{Y}} }_{h}= \frac{\sum_{i=1}^{{n}_{h}}\sum_{k=1}^{{m}_{hi}}{w}_{hik}{y}_{hik}}{\sum_{i=1}^{{n}_{h}}\sum_{k=1}^{{m}_{hi}}{w}_{hik}}$$

The estimate of average value of the variable in the state is as

$$\widehat{\overline{Y} }= \frac{\sum_{h=1}^{H}\sum_{i=1}^{{n}_{h}}\sum_{k=1}^{{m}_{hi}}{w}_{hik}{y}_{hik}}{\sum_{h=1}^{H}\sum_{i=1}^{{n}_{h}}\sum_{k=1}^{{m}_{hi}}{w}_{hik}}$$

The estimates of variance of the estimate of average value of the variable in the state is as

$$\widehat{V}\left(\widehat{\overline{Y} }\right)= \frac{\sum_{h=1}^{H}\sum_{i=1}^{{n}_{h}}\sum_{k=1}^{{m}_{hi}}{w}_{hik}\left({w}_{hik}-1\right){\left({y}_{hik}-\widehat{\overline{Y} }\right)}^{2}}{{\left(\sum_{h=1}^{H}\sum_{i=1}^{{n}_{h}}\sum_{k=1}^{{m}_{hi}}{w}_{hik}\right)}^{2}}$$

where ${y}_{hik}$ is the values of the variables corresponding to *k*-th household in the *i*-th selected village belonging to the *h*-th ACZ, ${w}_{hik}= {w}_{h}\times {w}_{hi}= \frac{{N}_{h}}{{n}_{h}} \times \frac{{M}_{hi}}{{m}_{hi}}$ is the survey weight of the *k*-th household in the *i*-th selected village belonging to the *h*-th stratum; ${N}_{h}$ is the total number of villages in the* h*-th ACZ; ${n}_{h}$ is the number of villages to be selected out of ${N}_{h}$ villages in the *h*-th ACZ; ${M}_{hi}$ is the total number of eligible farm households in *i*-th selected village in the *h*-th ACZ, and ${m}_{hi}$ is the number of eligible farm households to be selected in the *i*-th selected village in the *h*-th ACZ.

### Energy equivalents

Onion production requires various energy inputs, and based on the energy equivalents of the inputs and output, following parameters were calculated as:

$$\text{Energy Ratio }(\text{Energy Use Efficiency}) = \frac{\text{Energy output }(\text{MJ}/\text{ha})}{\text{Energy Input }(\text{MJ}/\text{ha})}$$

$$\text{Energy Productivity }= \frac{\text{Onion output }(\text{Kg}/\text{ha})}{\text{Energy Input }(\text{MJ}/\text{ha})}$$

$$\text{Specific Energy }= \frac{\text{Energy Input }(\text{MJ}/\text{ha})}{\text{Onion output }(\text{Kg}/\text{ha})}$$

$$\text{Net Energy }=\text{ Energy output }(\text{MJ}/\text{ha})-\text{Energy input }(\text{MJ}/\text{ha})$$

### Data envelopment analysis (DEA)

The DEA is a method of analysis used to measure the relative efficiency of a set of comparable units called decision-making units (DMUs) by using specific mathematical programming models^48^. Charnes et al. 1978^49^ developed the Charnes-Cooper-Rhodes (CCR) model, which exhibits constant returns to scale (CRS). Banker et al. 1984^50^ extended the CCR model to the Banker-Charnes-Cooper (BCC) model, which exhibits variable returns to scale (VRS). These models are centered on determining the most efficient farm households that can be used as a reference, with which the efficiency of the rest of the farm households is compared.

#### Technical efficiency and scale efficiency

Technical efficiency is the ability of DMUs to achieve maximum output from the given inputs^51^:

$$\text{Technical Efficiency }(\theta ) =\frac{\text{weighted sum of outputs}}{\text{weighted sum of inputs}}= \frac{\sum_{r=1}^{s}{u}_{r}{y}_{rj}}{\sum_{i=1}^{m}{v}_{i}{x}_{ij}}$$

To measure the relative efficiency of a DMU based on a series of all other DMUs, the model is structured as a fractional programming problem as follows^10^:

$$\text{maximize} \theta = \frac{\sum_{r=1}^{s}{u}_{r}{y}_{rj}}{\sum_{i=1}^{m}{v}_{i}{x}_{ij}}$$

$$\text{subject to constraint}: \frac{\sum_{r=1}^{s}{u}_{r}{y}_{rj}}{\sum_{i=1}^{m}{v}_{i}{x}_{ij}}\le 1$$

$${u}_{r}, {v}_{i}\ge 0$$

where n: number of DMUs in the comparison set; s: number of outputs; m: number of inputs, ${u}_{r}$: weighting of output ${y}_{r}$; ${v}_{i}$: weighting of input ${x}_{i}$; ${y}_{rj}$: values of outputs; ${x}_{ij}$: values of inputs.

Technical Efficiency $(\theta )$ = 1 indicates a technically efficient DMU whereas $\theta <1$ indicates technically inefficient DMUs. For each inefficient DMU, target input levels is calculated based on slack variables (${s}_{i}^{-}$) as^51^:

$$Input Value \left({\widehat{x}}_{io}\right)= \theta \times {x}_{io}- {s}_{i}^{-}$$

Scale efficiency $(SE)= \frac{{\theta }_{CRS}}{{\theta }_{vRS}}$. Scale efficiency less than one indicate scale inefficiency^52^. Data analysis in this study was carried out in R-software (R-4.4.2, https://cran.r-project.org/bin/windows/base/, https://www.rstudio.com/products/rstudio/download/) for generation of the estimates and “Benchmarking” package was used for DEA.

## Results and discussion

### Energy use pattern

The estimates of average energy consumption for all operations, along with the coefficient of variation, are presented in Table 1. Onions are typically transplanted to a main field that has been properly plowed to remove agricultural remains and soil clods, using a double plow, tyne cultivator, and rotavator. In this study, it was found that energy consumption during land preparation for the main field was 3336.33 MJ/ha, which accounted for 17% of the total input energy used in onion production. After transplanting, operations such as fertilizer application, weed management, and irrigation are required for efficient crop production. Onions are more susceptible to weed competition than other crops, primarily due to their slow growth during the early stages of development and inherent characteristics such as short stature, non-branching, and a shallow root system. Fertilizer application accounted for a significant share of energy input in onion production, at 43.84%, followed by irrigation (14.56%) and land preparation for the main field (16.13%). Fertilizer application is a significant input because it can significantly increase crop yield, resulting in better profits. Additionally, it helps the plant to grow stronger and healthier, making it more resistant to pests and diseases. These results parallel those of Phipps et al. 1976^53^, where fertilizers were identified as a major energy input, and Komleh et al. 2011^54^, who found that fuel and chemical fertilizers were the highest energy consumers in crops in the Guilan province of Iran. It is recommended that energy inputs in terms of fertilizers can be reduced by using liquid fertilizer, precision application of fertilizers, and spreading awareness among farm households for optimal application of fertilizer. In addition to this, applying fertilizers at the right time is important to coincide with the growth stages of the crop. This helps to ensure that the crop receives the appropriate amount of nutrients when it needs them, avoiding unnecessary waste.

**Table 1:** Operation-wise estimates of average energy input in onion production in Karnataka, India.

Operation	Estimate of average energy Input (MJ/ha)	Coefficient of variation (%)
Land preparation for main field	3336.33 (16.13%)	2.93
Sowing	488.78 (2.36%)	0.61
Manure application	1421.23 (6.87%)	0.77
Fertilizer application	9064.94 (43.84%)	0.97
Plant protection	1716.77 (8.30%)	13.37
Irrigation	3010.86 (14.56%)	3.69
Harvesting	557.68 (2.70%)	2.83
Detopping	601.15 (2.91%)	0.56
Grading	481.35 (2.33%)	0.56

The energy consumption for plant protection (1716.77 MJ/ha) in onion crop production is relatively low compared to other operations because onions are less susceptible to pests and diseases compared to other crops, and therefore require less energy input for plant protection. However, the energy input for plant protection can vary depending on the pest and disease pressure in a particular area, and the type of pesticides and application methods used. The use of integrated pest management (IPM) techniques, such as biological control and crop rotation, can help to reduce the energy input for plant protection while maintaining crop yields and quality. Regarding irrigation, the use of modern irrigation techniques such as drip and micro sprinkler irrigation can significantly reduce energy input and water use while maintaining crop yields. It is important to promote the adoption of these techniques among farmers to improve the sustainability of onion crop production. The operations like harvesting (557.68 MJ/ha), detopping (601.15 MJ/ha), and grading (481.35 MJ/ha) are labor-intensive and require significant amounts of energy. Mechanization of these operations through the use of appropriate technologies can help to reduce the energy input and labor requirements, while increasing efficiency and productivity. Ozbek et al. (2021)^55^ reported that the total energy inputs for onion cultivation were 22,463.52 MJ/ha. Of this, chemical fertilizers accounted for 60.43%, human labor contributed 15.34%, and seed energy made up 1.01%.

**Fig. 2:**
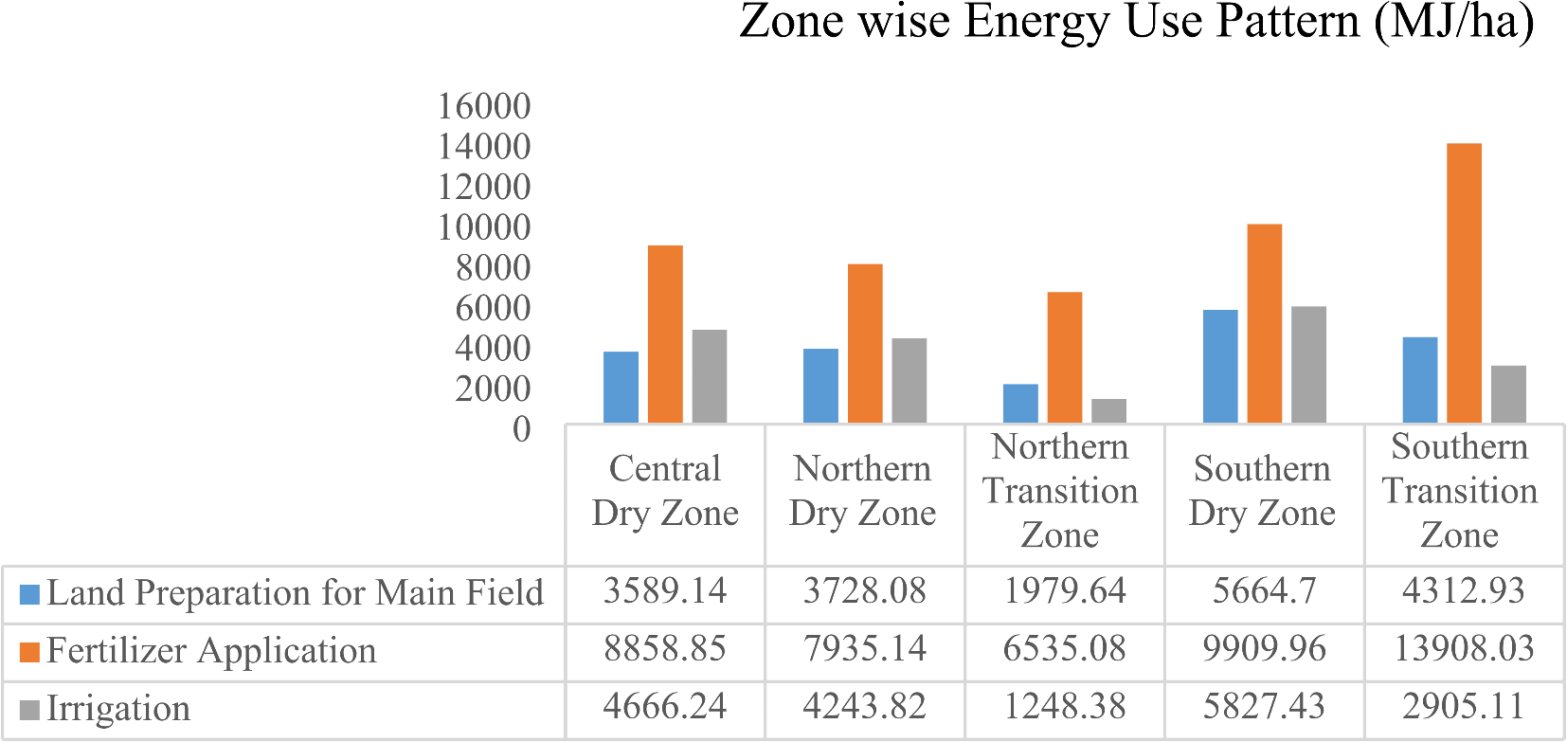
Operation and zone-wise average energy consumption estimates of onion production.

**Fig. 3:**
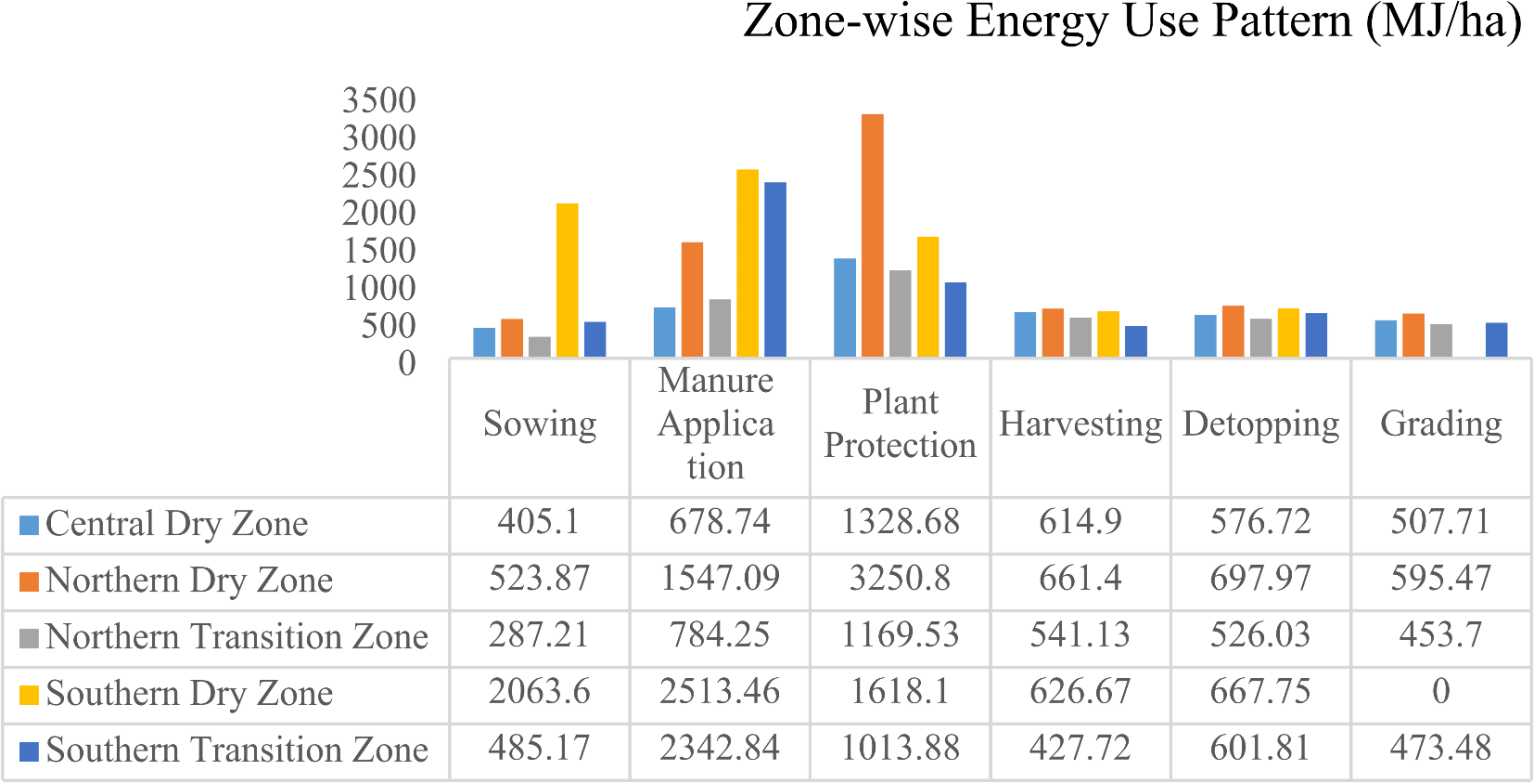
Operation and zone-wise average energy consumption estimates of onion production.

Operation and zone-wise average energy consumption estimates of onion production is also presented in Figs. 2 and 3. Five out of ten agro-climatic zones of Karnataka were considered for conducting an energy audit survey of the onion crop. These five agro-climatic zones were central dry zone, northern dry zone, northern transition zone, southern dry zone, and southern transition zone.

The energy consumption for land preparation for main filed was maximum in southern dry zone (5664.7 MJ/ha), followed by southern transition zone (4312.93 MJ/ha), and northern dry zone (3728.08 MJ/ha). The northern transition zone required lowest energy for this operation, with a consumption of 1979.64 MJ/ha. The energy input in term of fertilizer application is highest among all operations particularly in southern transition zone (13908.03 MJ/ha). The southern dry zone also showed high energy consumption for this operation (9909.96 MJ/ha). The northern transition zone required the least energy for fertilizer application, with a consumption of 6535.08 MJ/ha. Energy input in terms of irrigation was highest southern dry zone (5827.43) followed by central dry zone (4666.24 MJ/ha), northern dry zone (4243.82 MJ/ha), southern transition zone (2905.11 MJ/ha), and northern transition zone (1248.38 MJ/ha).

The energy input in term of sowing was maximum in southern dry zone (2063.6 MJ/ha) and lowest in central dry zone (405.10 MJ/ha). This operation requires minimal energy in most zones excepts southern dry zone and southern transition zone. There was considerable variation in energy input in terms of manure application across the zones, with the southern dry zone having the highest energy consumption at 2513.46 MJ/ha, while the northern transition zone recorded the lowest energy use at 784.25 MJ/ha. The energy use in plant protection was highest in northern dry zone (3250.80 MJ/ha) and lowest in southern transition zone (1013.88 MJ/ha).

The energy use in harvesting, detopping and grading was highest in northern dry zone i.e. 661.40 MJ/ha, 697.97 MJ/ha, 595.47 MJ/ha respectively and lowest in southern transition zone (for harvesting) and northern transition zone for detopping and grading. Energy inputs varied across operations in each zone which might be influenced by factors like soil fertility, crop types, or climate conditions. For example, southern dry zone required more energy input, while northern transition zone uses the least energy in most of the operations.

The estimates of source-wise consumption of energy input was also computed which indicated that chemical fertilizers contribute 44.25% of the total energy input, followed by diesel (18.82%) and manual operations (15.01%). Harvesting, detopping, and grading in onion crops are mainly done manually. Farm households usually apply chemical fertilizers in large quantities to increase production. FYM and mechanical activities consume 6.38% and 1.18% of the total energy input, respectively. Onions require a high level of nutrients for better production, and chemical fertilizers contain higher amounts of nutrients than FYM. Furthermore, chemical fertilizers are readily available, more cost-effective, and easier to use than FYM. For these reasons, chemical fertilizers are generally preferred over FYM for onion production in India.

Direct energy (Human labor, fossil fuels, and electricity) accounted for 33.97%, while indirect energy (machinery, FYM, fertilizers, and seeds) accounted for 66.03% of the total energy used in onion production in the study area. These findings are consistent with those of Ozbek et al. 2021^55^. Renewable energy sources accounted for only 15.4% of the energy used in onion production, while non-renewable energy sources accounted for 84.6%. This indicates a significant sustainability issue in the context of global warming, as the heavy use of non-renewable energy sources can have a negative impact on environmental quality by producing a large amount of greenhouse gases^56^. Therefore, it is crucial to increase the utilization of renewable energy sources to conserve energy without harming the environment. Renewable energy sources such as solar energy, wind energy, geothermal energy, and hydroelectric power can also enhance national energy security.

Energy Ratio (Energy Use Efficiency), Energy Productivity, Specific Energy, and Net Energy of onion production is presented in Table 3. The average yield of onion in the state of Karnataka, India was estimated to be 7619.66 kg/ha with maximum yield in southern transition zone (9815.80 kg/ha) and minimum in northern transion zone (5044.13 kg/ha) Table 2 indicates that the energy ratio was greater than one, indicating that the energy inputs used by farm households in onion crop production were efficient in all zones except for the southern dry zone. Therefore, it is suggested that energy use efficiency can be increased by reducing energy inputs, using energy-efficient appliances, utilizing renewable energy sources, and promoting behavioral changes towards sustainable agriculture, which could bring about improvements in the future. The average energy productivity in Karnataka was 0.368 kg/MJ. This indicated that 0.368 units output was obtained per unit energy. The net energy of onion was positive for all the agroclimatic zones except southern dry zone. In this zone, net energy of onion was negative (− 3796.48 MJ/ha), inferring that energy is being lost in onion production in southern dry zone. Omid et al. 2011^45^ also got negative net energy indicating that energy is being lost for cucumber production in Iran.

**Table 2:** Energy output–input ratio and forms in onion production.

Zones	Central dry	Northern dry	Northern transition	Southern dry	Southern transition	Karnataka
Onion crop yield (Kg/ha)	6705.25	9605.17	5044.13	6350.00	9815.80	7619.66
Energy ratio	1.248	1.637	1.474	0.869	1.465	1.456
Energy productivity (Kg/MJ)	0.316	0.414	0.373	0.220	0.371	0.368
Specific energy (MJ/Kg)	3.166	2.414	2.681	4.549	2.697	2.714
Net energy (MJ/ha)	5273.07	14,775.98	6409.43	− 3796.48	12,321.05	9433.81

### Data envelopment analysis (DEA)

Data Envelopment Analysis (DEA) was conducted to distinguish between efficient and non-efficient farm households in onion production in Karnataka based on their energy inputs. These inputs include human labor (hr), machinery (MJ), Electricity (kW/h), Nitrogen (kg), Phosphorus (kg), Potassium (kg), Weedicide (kg), Diesel (l) and Seed (kg). Human labor accounts for both male and female workers, whether owned or hired, involved in all stages of crop production, including land preparation, irrigation, fertilizer application, weeding, harvesting, grading, detopping, and more. Machinery (MJ) includes equipment such as tractors, disc ploughs, cultivators, rotovators, seed drills, sprayers, and motors. The electricity (kWh), weedicide (kg), diesel (liters), and seed (kg) values represent the total amount of each input used during the production cycle. The quantities of nitrogen (kg), phosphorus (kg), and potassium (kg) are based on the fertilizer compositions applied throughout the crop’s growth. Omid et al. (2011) also employed these inputs in their DEA analysis while investigating the energy use patterns and benchmarking of selected greenhouses in Iran.

Out of the 600 farm households studied, 241 (40.17%) had a pure technical efficiency score of one. Among these, 43 farm households had a technical efficiency score of 1, indicating that they were efficiently using energy inputs compared to other farm households in the study area. These efficient farm households (DMUs) could serve as a benchmark for inefficient DMUs. Several studies have used DEA to identify efficient DMUs in agriculture, such as rice^57^ and cucumber^58^, but research related to crops like onion is limited.

The technical efficiency, pure technical efficiency, and scale efficiency had average values of 0.77, 0.92, and 0.84, respectively. The average technical efficiency for inefficient farm households was 0.77. This suggests that if these units were performing on the frontiers i.e. maximizing the performance of these DMUs, the same level of output could be produced with 77% of the resources, leading to a potential saving of 23% of total resources. This implies that there is considerable scope for improving the energy use efficiency of inefficient farm households, which will have a direct impact on their profitability and sustainability through agriculture. The average scale efficiency was 0.84, indicating that some energy could be saved from various sources if inefficient farm households use their resources effectively without changing their technology of farm management. A farm household with a technical efficiency less than one is using more energy than required from the various sources. These results suggest the need for sensitization of inefficient farm households towards the efficient use of inputs during crop production to help achieve sustainability in agricultural production. It is also desirable to suggest realistic levels of energy to be used from each source for each inefficient grower to avoid energy waste while maintaining profitability. Inefficient energy use or unnecessary use of excessive energies may lead to more greenhouse gas emissions, which will contribute to the problem of global warming. Omid et al. 2011^45^ applied data envelopment analysis to assess productivity performance of 18 cucumber producers. Their findings indicated that 12 out of the 18 DMUs were efficient. The average values for pure technical efficiency, technical efficiency and scale efficiency were 0.972, 0.879 and 0.9, respectively. Among the inefficient DMUs, the mean technical efficiency was 91.5%, suggesting that these units could achieve the same level of output using only 91.5% of their current resources if they operated on the efficiency frontier. However, similar studies on onion production are limited in the literature. Table 3 shows the amount of greenhouse gas emissions in kgCO_2_ equivalent/ha of land during the production of onion.

**Table 3:** Greenhouse Gas (GHG) Emissions in Onion Production in Karnataka, India.

Inputs	Average estimates of Inputs used (MJ/ha )	GHG coefficient (kg CO_2_eq/MJ)	GHG emissions (kgCO_2_eq/ha)
Human labor (hr.)	568.18	0.700^55^	397.73
Machinery (MJ)	98.9	3.320^59^	328.35
Electricity (kW/h)	100.99	0.820^59^	82.81
FYM (kg)	1780.87	0.006^59^	10.69
Nitrogen (kg)	35.19	1.300^60^	45.75
Phosphorus (kg)	43.31	0.200^60^	8.66
Potassium (kg)	22.56	0.200^60^	4.51
Weedicide (kg)	0.45	6.300^60^	2.84
Diesel (l)	26.21	0.940^60^	24.64
Seed (kg)	20.8	7.630^55^	158.71

These results indicate that farmers with a technical efficiency less than one are producing more CO_2_ than the optimal amount, which can have negative long-term impacts on the environment. The highest GHG emissions in onion cultivation in the study area was from human labor, contributing 397.73 kg CO_2_eq/ha, highlighting the labor-intensive nature of the process. Machinery use (328.35 kg CO_2_eq/ha) and diesel consumption (24.64 kg CO_2_eq/ha) also play a major role, indicating the impact of mechanization and fossil fuel dependence. Fertilizer application, particularly nitrogen (45.75 kg CO_2_eq/ha), adds to emissions, while phosphorus (8.66 kg CO_2_eq/ha) and potassium (4.51 kg CO_2_eq/ha) have a comparatively lower impact. Overall, optimizing labor efficiency, adopting renewable energy sources, improving fertilizer management, and enhancing mechanization efficiency could help reduce GHG emissions in onion farming. The findings can also help policy planners identify areas for improvement in mitigating the problem of global warming in crop production. Ozbek et al. (2021)^55^ reported that total greenhouse gas (GHG) emissions from onion cultivation were 2920.73 kgCO_2_eq/ha, with human labor contributing the largest share at 42.13%. Other studies have reported GHG emissions for different crops: Karaağaç et al.^61^ for chickpea cultivation, Baran et al.^62^ reported for almonds, and Eren et al.^63^ for sunflower cultivation.

Table 4 displays the average target input and contribution input to saving (%) for different sources. Target input levels for inefficient farm households can be calculated by multiplying the input value with an optimal efficiency score and then subtracting slack amounts from this amount^51^.

**Table 4:** Target Input (MJ/ha) from Different Sources for Inefficient Onion Farm Households of Karnataka, India.

Inputs	Present use (MJ/ha)	Target use (MJ/ha)	Energy saving (MJ/ha)	Contribution input to saving (%)
Machinery	254.631	147.339	107.292	1.48
Human labor	3525.51	2577.83	947.679	13.03
Diesel	4737.59	3304.43	1433.16	19.7
Electricity	3669.31	2102.38	1566.93	21.54
FYM	1339.32	631.041	708.28	9.74
Fertilizers	8272.91	5823.69	2449.22	33.67
Seed	68.266	7.65433	60.6117	0.83

From Table 4, it can be observed that the maximum contribution to the total energy savings is 33.67% from fertilizers, followed by electricity (21.54%) and human labor (13.03%). The study indicates that there is considerable potential for saving energy by adopting efficient cultivation practices such as the use of conservation agriculture, Nano-fertilizers, solar-operated tractors, bio-diesels, solar pumps, and windmills for energy inputs in terms of electricity. The efficient utilization of energy sources will lead to fewer greenhouse gas emissions from agricultural practices for crop production and will aid in maintaining pollution levels. It will help mitigate the problem of global warming and promote the implementation of renewable energy sources such as solar or wind energy as an alternative to conventional sources for achieving the UN’s SDG 7.0 in major developing countries like India.

## Conclusions

In this study, a sampling methodology was proposed to estimate energy audit and CO_2_ emissions associated with various crop management practices during onion production in Karnataka, India. Data analysis revealed that fertilizer application, irrigation, and land preparation were the major energy-intensive practices in onion production in the region. The substantial use of non-renewable energy inputs like fertilizers and petrol for farm mechanization may lead to environmental problems such as global warming, nutrient loading, and groundwater pollution. Data Envelopment Analysis (DEA) indicated that only 7% of onion farm households in Karnataka were using energy inputs efficiently. The average technical efficiency of inefficient farm households was found to be 0.77, implying that around 23% of resources might be saved by adopting more efficient practices. The study also found that human labor, which is relatively cheaper in India compared to other countries, resulted in the highest emissions of kgCO_2_ equivalent/ha of land, followed by farm machinery, seed, and electricity for irrigation. Based on the study findings, it can be concluded that there is significant potential for energy conservation through the efficient use of resources and the adoption of renewable energy systems such as wind or solar energy for crop production in developing countries like India. This would help in mitigating the problem of global warming and reducing GHG emissions from agricultural fields during crop management. Sensitizing farm households about the adverse effects of excessive CO_2_ emissions and promoting the use of bio fertilizers, bio control agents, bio-diesels, and solar-operated machinery would also be necessary. Introduction of high-yielding improved varieties can also reduce the use of chemicals and irrigation, thereby increasing energy use efficiency and energy productivity for onion crop in India.

## Data Availability

The datasets generated during and/or analyzed during the current study are not publicly available as it belongs to Indian Council of Agricultural Research-All India Coordinated Research Project on Energy in Agriculture and Allied Industries but may be made available from the corresponding author on reasonable request.
